# EFFECT OF COPAIBA OIL IN INTESTINAL MUCOSA OF RATS SUBMITTED TO HYPOVOLEMIC SHOCK

**DOI:** 10.1590/0102-672020190001e1451

**Published:** 2019-10-21

**Authors:** Renan Kleber Costa TEIXEIRA, Felipe Lobato da Silva COSTA, Faustino Chaves CALVO, Deivid Ramos dos SANTOS, Edson Yuzur YASOJIMA, Marcus Vinicius Henriques BRITO

**Affiliations:** 1 Laboratory of Experimental Surgery, Faculty of Medicine, State University of Pará, Belém, PA, Brazil.

**Keywords:** Mesenteric vascular disease, Small intestine, Medicinal plants, Rats, Doença vascular intestinal, Intestino delgado, Plantas medicinais, Ratos

## Abstract

**Background:**

Hypovolemic shock is a common disease in polytrauma patients and may develop ischemia in various organs, increasing morbidity and mortality. The bowel is usually most affected by this condition.

**Aim:**

To evaluate the effects of copaiba oil on the intestinal mucosa’s injury of rats submitted to hypovolemic shock.

**Method:**

Fifteen rats were divided into three groups: sham - simulated surgery; ischemia - animals submitted to hypovolemic shock; and copaiba - animals submitted to hypovolemic shock previously treated with copaiba oil. Mean blood pressure, arterial blood gas after shock induction, degree of intestinal lesion and villus length were evaluated.

**Results:**

The sham presented the lowest values of lactate and PaCO_2_ and the highest values of mean arterial pressure, pH and bicarbonate in relation to the other groups. The degree of mesenteric lesion was zero in the sham group; 3.00±1.00 in the ischemia group; and 3.00±0.71 in the copaiba group. The villus length was 173.60±8.42 in the sham, 142.77±8.33 in the ischemia and 143.01±9.57 in the copaiba group. There was a significant difference between the sham and the other groups (p<0.05); however, there not significant difference between groups Ischemia and copaiba.

**Conclusion:**

Administration of copaiba oil did not reduce the intestinal mucosa lesion of rats after hypovolemic shock.

## RESUMO

**Racional:**

O choque hipovolêmico é situação comum em pacientes politraumatizados, podendo acarretar isquemia de vários órgãos, aumentando a morbimortalidade. O intestino é geralmente um dos órgãos mais afetados por essa condição.

**Objetivo:**

Avaliar os efeitos do óleo de copaíba na lesão da mucosa intestinal de ratos submetidos ao choque hipovolêmico.

**Métodos:**

Quinze ratos foram distribuídos em três grupos: Sham - operação simulada; isquemia - submissão ao choque hipovolêmico; e copaíba - submissão ao choque hipovolêmico previamente tratados com óleo de copaíba. A pressão arterial média, a gasometria arterial após a indução do choque, o grau da lesão intestinal e o tamanho das vilosidades foram avaliados.

**Resultados:**

O grupo sham apresentou os menores valores de lactato e PaCO_2_ e os maiores valores de pressão arterial média, pH e bicarbonato em relação aos demais grupos. O grau de lesão mesentérica foi de zero no sham; 3,0±1,00 no grupo isquemia; e 3,0±0,71 no da copaíba. O comprimento dos vilos foi de 173,60±8,42 no grupo sham, 142,77±8,33 no da isquemia e 143,01±9,57 no da copaíba. Houve diferença significante entre o grupo sham e os demais grupos (p<0.05); contudo, não houve diferença estatística entre os grupos submetidos ao choque hipovolêmico.

**Conclusão:**

A administração do óleo de copaíba não reduziu a lesão da mucosa intestinal de ratos submetidos ao choque hipovolêmico.

## INTRODUCTION

Trauma is one of the leading causes of death in the world, especially in young adults^12^. Several are the mechanisms that lead to severe organ dysfunctions^16^, and hypovolemic shock is one of the most common. It causes ischemia of several organs, especially kidneys and splanchnic territory^3^ ^,^ ^15^.

The bowel is sensitive to ischemia because of its high metabolic activity^19^; resulting in worsening of wound healing after anastomoses^5.7^, increased fistulas tax, and difficulty in adequate nutrients’ absorption, causing malnutrition, anemia and diarrhea. It is one of the main sources of bacteria causing sepsis, which happens with the breakdown of the intestinal barrier allowing wide bacterial translocation^17^.

Several alternatives were evaluated aiming to reduce ischemic injury in the intestinal mucosa. For this, hypertonic saline solutions^13^ and hydrocortisone^23^ were used during volume resuscitation; medications such as pentoxifylline^18^ and N-acetyl-cysteine^1^; probiotics^22^; and enteral nutritional therapy^14^. However, these methods had dubious or unhelpful effects.

Copaiba oil originates from the sap of copaifera trees (family Fabaceae) and presents proven actions^6^ ^,^ ^24^ ^,^ ^25^ as anti-inflammatory, healing and antioxidant. This oil was tested on colitis induced^20^ by acetic acid in rats and had a positive anti-inflammatory effect. In addition, in a study evaluating the effects of it on abdominal sepsis by cecal ligation and puncture^17^, there was an increase in overall survival with a lower degree of lung injury and lower levels of oxidative stress.

Thus, this study aims to evaluated the effects of copaiba oil on the intestinal mucosa of rats submitted to hypovolemic shock.

## METHODS

This study was approved by the Ethics Committee for the Use of Animals of the State University of Pará - UEPA, protocol 11/11. Fifteen male Wistar rats (*Rattus norvegicus*) obtained from the Animal Colony of the Experimental Surgery Laboratory of UEPA were used. They weighed 200-250 g and were kept in a controlled environment with food and water ad libitum.

The animals were randomly assigned into three study groups (n=5): 1) sham group (SG): same surgical procedure as in remaining groups was performed, but no hypovolemic shock was induced; 2) ischemic group (CG): animals submitted to hypovolemic shock; 3) copaiba group (CG): animals submitted to hypovolemic shock previously treated with copaiba oil.

The CG animals received copaiba oil (*Copaifera officinalis*) by gavage at the dose of 0.63 ml/kg once daily for seven days^27^ prior to the induction of hypovolemic shock. The spectrophotometric analysis of the oil is described in Table 1. The SG and IG animals received 0.9% saline in the same proportions as copaiba oil.

**TABLE 1:** Composition of copaiba (*Copaifera officinalis*) oil used

Constituents	%
Limonene	0,24
α-copaene	0,10
7-*epi*-sesquithujene	0,25
cyperene	0,42
*cis*-α-bergamotene	0,15
β-caryophyllene	10,00
trans-α-bergamotene	18,32
epi-β-santalene	0,05
α-humulene	3,02
γ-curcumene	0,24
mw=204	2,01
α-zingiberene	0,21
β-bisabolene	52,03
(*Z*)-α-bisabolene	2,90
β-sesquiphelandrene	2,31
mw=204	3,98
(E)-γ-bisabolene	0,20
caryophyllene alcohol	0,05
caryophyllene oxide	0,33
β-atlantol	0,12
epi-β-bisabolol	0,21
epi-α-bisabolol	0,53

All surgical procedures were performed under anesthesia (ketamine - 70 mg/kg and xylazine - 10 mg/kg, intraperitoneally). Once the anesthetic plane was confirmed, a median cervicotomy and dissection of the right region were performed until identification and isolation of the right common carotid artery under magnification of videomicrosurgery system^2^. The artery catheterization was performed with a 24º G jelco, previously heparinized; then 30% of the volume of the animal was drained (based on the formula: “total volume (ml)=animal weight (grams) x0.06”)^26^ using a glass syringe lasting 10 min, thus inducing hypovolemic shock. After the collection of blood, the heparinized jelco was kept for a new blood collector.

The animals were kept in a warm environment at 36º C and resuscitated volumetrically with 0.9% saline at a dose of 10 ml/kg subcutaneously. Fifty minutes after the shock induction, the mean arterial pressure was measured and 0.1 ml of blood was collected for arterial blood gas analysis^23^ through the fixed jelco in the right common carotid artery. After collection, was performed the ligation of in both artery stumps with 4-0 silk, and the skin was sutured with 5-0 nylon.

After this procedure, the animals were followed for 72 h. At the end of this period, euthanasia was performed by intraperitoneal injection of high dose xylazine. Immediately after, a 2 cm ileum sample distant 3 cm from the cecum valve was harvested. This fragment was fixed in 10% buffered formaldehyde, and underwent histological processing; the slides were stained with hematoxylin-eosin and examined under light microscopy to determine the degree of injury based on the scale of Chiu et al^7^ and villous length measurements were done by measuring ten well-oriented villous^5^ (cut across its length) in each of the animals.

### Statistical analysis

BioEstat^®^ software 5.4 (Belém, PA, Brazil) was used to perform statistical analysis. ANOVA test was used to compare villous length and gasometrical parameters; Kruskal-Wallis test, to compare the histopathological results. A value of p<0.05 was used for significance of the comparisons between the three groups.

## RESULTS

During the procedure and follow-up no animal died or resuscitation maneuvers were performed. The mean of arterial pressure was 75.00±1.20 mmHg on SG, 42.20±2.68 mmHg on IG and 42.60±1.67 mmHg on CG. There was a significant difference between SG than IG and CG (p<0.01).

In relation to the gasometric analysis (Table 2), the SG presented the lowest values of lactate and PaCO_2_ and the highest values of pH and bicarbonate in relation to the IG and CG groups (p<0.05). There was no significant difference between the IG and CG groups.

**TABLE 2:** Mean and standard deviation of gasometric parameters according the groups

Parameter	Sham^*^	Ischemia	Copaiba
pH	7.39±0.02	7.08±0.06	7.11±0.04
PaCO_2_	54.00±3.80	65.20±3.70	63.60±4.77
HCO_3_	25.80±1.48	16.40±3.57	16.20±2.28
Lactate	1.42±0.44	7.20±1.64	7.60±0.54

p<0,05 sham vs. other groups (ANOVA)

The mean score of intestinal injury is demonstrated on Table 3. Sham group showed a normal mucosa, and there was significant difference between this group to all others who underwent hypovolemic shock; among the Ischemic and copaiba groups there was no significant difference (p>0.05). In relation to the villous length (Table 3), there was difference between the sham group and the others groups (p<0.05); however, there was no difference between the ischemic and copaiba groups (p>0.05).

**TABLE 3:** Mean and standard deviation of intestinal injury score and villous length according the groups

Parameter	Sham	Ischemia	Copaiba
Intestinal injury	0.00±0.00*	3.00±1.00	3.00±0.71
Villous length	173.60±8.42**	142.77±8.33	143.01±9.57

* p<0,05 sham vs. other groups (ANOVA); **p<0,05 sham vs. other groups (Kruskal-Wallis)

## DISCUSSION

The use of medicinal plants is a practice that has been stimulated by the World Health Organization and the current guidelines of the Unified Health System, given the difficulty of the population in the acquisition of medicines; however, there is the possibility of recognition of new active principles that can be added to the current therapeutic arsenal. However, there are few scientific reports that support the use of most medicinal plants, and little is known about their possible side effects^6^ ^,^ ^20^ ^,^ ^24^ ^,^ ^25^ ^,^ ^27^.

Copaiba oil has several proven scientific effects such as healing, antibiotic, anti-inflammatory and antioxidant ^6^ ^,^ ^17^ ^,^ ^20^ ^,^ ^24^ ^,^ ^25^ ^,^ ^27^. The ischemia and reperfusion syndrome were tested in vascular occlusion in experimental models in renal^4^, hepatic^9^, skin^10^ and mesenteric ischemia^21^, and in all scenarios presented lower cellular damage, mainly due to the high levels of β-caryophyllene.

In the present study, based on the evaluated parameters, the copaiba oil had no beneficial effect, since it had similar results in the groups where it was used. Seven-day prophylaxis using it has shown in several earlier studies better healing^6^ ^,^ ^20^ ^,^ ^24^ ^,^ ^25^ ^,^ ^27^ and protection against ischemic injuries^4^ ^,^ ^9^ ^,^ ^10^ ^,^ ^21^; however, none evaluated the action of the oil in a hypovolemic shock model.

The mean arterial pressure in both groups submitted to hypovolemia was the same, as initially predicted, due to the low influence of treatment on the general volemia of the animal. However, there was no change in the gasometric profile and intestinal histological lesion, in contrast to that found in most studies evaluating copaiba oil in ischemia and reperfusion of specific organs. Comelli Junior^8^ also did not identify alteration in the healing of intestinal anastomoses in rats treated with copaiba oil.

The high degree of systemic inflammation caused by hypovolemic shock must have been one of the factors that influenced the result, reducing to almost imperceptible levels the improvement caused by copaiba oil. The use of high doses (200-400 mg/kg)^11^ might have better effects.

It is important to note that the negative result of this research does not indicate a failure. The acquisition of knowledge with null results helps other researchers not to repeat the same method, and the publication of these results is valid because it saves time and funding, and also causes modifications that can lead to positive results with another study design.

## CONCLUSION

Administration of copaiba oil did not reduce the intestinal mucosa lesion of rats after hypovolemic shock.
